# Host ecology drives frog skin microbiome diversity across ecotone in South-Central North America

**DOI:** 10.3389/frmbi.2023.1286985

**Published:** 2023-11-07

**Authors:** Sierra N. Smith, Jessa L. Watters, Cameron D. Siler

**Affiliations:** ^1^ Sam Noble Oklahoma Museum of Natural History, Norman, OK, United States; ^2^ School of Biological Sciences, University of Oklahoma, Norman, OK, United States

**Keywords:** 16S rRNA, amphibians, ecoregion, ecology, Oklahoma, skin microbiome

## Abstract

Anurans (frogs and toads) are an ecologically diverse group of vertebrate organisms that display a myriad of reproductive modes and life history traits. To persist in such an expansive array of habitats, these organisms have evolved specialized skin that is used for respiration while also protecting against moisture loss, pathogens, and environmental contaminants. Anuran skin is also colonized by communities of symbiotic microorganisms, and these skin microbiota serve critical roles in numerous processes associated with anuran host health and persistence such as pathogen resistance and immunity. However, gaps remain in our understanding of the environmental and evolutionary processes that shape frog skin microbial communities. Here, we combined existing anuran disease data with 16S rRNA skin microbial inventories to elucidate the roles that geographic location, host evolutionary history, host ecology, and pathogen presence play in the microbial community assemblage of five co-distributed frog host species in Oklahoma. These focal species possess distinct ecological preferences: aquatic, semi-aquatic, and arboreal, and our results indicate that host ecology is the primary driver of frog skin microbial community structure. Additionally, compositional differences were observed among select host species based on geographic location, but this was not consistent among all five frog species. We did not find evidence of phylogenetic signal among our samples and results from the Classification and Regression Tree Analysis revealed that the presence of the amphibian pathogen *Batrachochytrium dendrobatidis* and the severity of infection were not drivers of skin microbiome differences among our focal host species. Results from this comparative study contribute to our growing understanding of the environmental and host-associated drivers of skin microbial community assemblage and represents one of the first studies on landscape-level variation in skin microbial communities among North American frogs.

## Introduction

Representing one of the five major clades of terrestrial vertebrates on the planet, amphibians (anurans, salamanders, and caecilians) display an astonishing diversity of life history traits (Vitt and Caldwell, 2013; Crump, 2015; Bardua et al., 2021). Their reproductive diversity (e.g., biphasic tadpole metamorphosis, direct development, etc.) and specialized ecomorphological adaptations (e.g., webbed toes, enlarged toe pads, etc.) have enabled amphibians to diversify across a near complete spectrum of environments on the planet, including fossorial, terrestrial, arboreal, aquatic, and riparian habitats (Afonso and Eterovick, 2007; Gómez and Lires, 2019; Engelkes et al., 2020; Bardua et al., 2021). To persist in such an expansive array of environments, amphibians have evolved specialized skin that serves as an essential respiratory organ and performs numerous physiological functions, including ion transport and osmoregulation, while also acting as a defensive barrier against harmful environmental and pathogenic agents (Duellman and Trueb, 1994; Lillywhite, 2006; Campbell et al., 2012; Kueneman et al., 2014; Wake and Koo, 2018; Varga et al., 2019). However, this same unique morphological feature also makes amphibians highly sensitive to instability in local environments, such as habitat modification and degradation, which has led to their recognition as bioindicators of ecosystem health (Wake, 1991; Blaustein et al., 1994; Houlahan et al., 2000; Ethier et al., 2021) and has contributed to alarming patterns of population declines on a global scale (Ford et al., 2020).

Currently, a staggering 41% of IUCN-assessed amphibian species are threatened with extinction (IUCN, 2023), with global population declines linked to a variety of factors, from pollution and invasive species to climate change and human-mediated habitat modification and destruction (Allentoft and O’Brien, 2010; Campbell Grant et al., 2020; Ford et al., 2020; Green et al., 2020). Furthermore, global declines have been exacerbated by growing threats posed by amphibian infectious diseases, including fungal and viral pathogens (Carey et al., 1999; Daszak et al., 1999; Greenberg and Palen, 2019; Scheele et al., 2019). In particular, *Batrachochytrium dendrobatidis* (*Bd*), a fungal pathogen that causes chytridiomycosis (often referenced as chytrid), has been especially devastating to anurans (frogs and toads), the most diverse of the three extant orders of amphibians with more than 7,500 species making up 88% of all amphibian diversity (AmphibiaWeb, 2023). To date, *Bd* infection has contributed to large-scale population declines of numerous species, as well as the confirmed or presumed extinction of more than 80 lineages to date (Scheele et al., 2019). Given that frogs and toads are essential members of both aquatic and terrestrial ecosystems (Whiles et al., 2006; Hocking and Babbitt, 2014), an improved understanding of the factors impacting anuran health, persistence, and immunity is needed for effective identification and mitigation of threats posed by rapid environmental change, habitat modification, and emerging pathogens.

Recently, a growing focus on the symbiotic microbial communities of host organisms, referred to as microbiomes, has revealed their fundamental roles in numerous processes associated with vertebrate host health, including digestion, nutrient acquisition, metabolism, immunity, development, and behavior (Ley et al., 2008; McFall-Ngai et al., 2013; McFall-Ngai, 2014; Alberdi et al., 2016; Colston and Jackson, 2016; Arizza et al., 2019; Rollins-Smith, 2020; Sehnal et al., 2021). Certain gut microbiota been shown to improve host metabolic activity in response to harsh environmental conditions (Li et al., 2019), with others often altering their composition and gene-expression patterns in response to physiological changes imposed by the host organism, underscoring the important role microbiomes may play in promoting host adaptation (Alberdi et al., 2016). Anuran skin microbiomes also serve a critical role as a protective barrier against pathogens (Harris et al., 2006; Harris et al., 2009; Becker et al., 2011; Kueneman et al., 2016; Ross et al., 2019; Rebollar et al., 2020; Jani et al., 2021). For example, metabolites produced by members of the frog skin microbiome have been shown to inhibit *Bd* zoospore development (Brucker et al., 2008; Loudon et al., 2014a; Walke and Belden, 2016). Furthermore, previous research has found that the presence of anti-fungal microbiota, such as *Janthinobacterium lividum*, on the skin of uninfected frogs reduced morbidity and mortality when the organisms were exposed to *Bd* (Harris et al., 2009; Walke and Belden, 2016). Unfortunately, despite the recognition that skin microbiome composition is influenced by many host-associated and environmental factors (Kueneman et al., 2014; Bletz et al., 2017; Carda-Diéguez et al., 2017; Prado-Irwin et al., 2017; Chiarello et al., 2018; Sehnal et al., 2021), little remains known about the patterns and the degree with which skin microbial communities change in response to environmental variation, particularly among diverse anuran hosts.

Studies to date indicate that anuran skin microbiomes are likely shaped by a number of factors such as host evolutionary history (McKenzie et al., 2012; Kueneman et al., 2014; Walke et al., 2014), host ecology (Bletz et al., 2017), and developmental stage (Kueneman et al., 2014; Jiménez et al., 2019). Additionally, local environment (Varela et al., 2018; Ellison et al., 2019; Rebollar and Harris, 2019), geographic location (Kueneman et al., 2014; Belden et al., 2015; Rebollar et al., 2016; Bletz et al., 2017), and the prevalence of emerging infectious disease (i.e. *Bd* infection; Rebollar et al., 2016; Familiar-López et al., 2017; Bell et al., 2018; Knutie et al., 2018; Rebollar and Harris, 2019; Jani et al., 2021) have been shown to influence anuran skin microbiome diversity. Although previous studies have compared the skin microbiomes of host communities from different sites (Kueneman et al., 2014; Belden et al., 2015; Rebollar et al., 2016; Bletz et al., 2017; Medina et al., 2017), few have systematically sampled frog skin microbiomes across environmental gradients (Bletz et al., 2017; Medina et al., 2017). Yet, climatic variation across ecotones is known to impact anuran community assembly through processes such as environmental filtering (Cortés-Gómez et al., 2013; Díaz-García et al., 2017; Álvarez-Grzybowska et al., 2020), implying that there are major gaps in our understanding of how large-scale climatic and ecological variation may impact anuran skin microbiomes, and in turn, host survival and persistence. Additionally, many anuran skin microbiome studies compare only a few (i.e., 1–3) host species (McKenzie et al., 2012; Kueneman et al., 2014; Walke et al., 2014; Varela et al., 2018). As such, there exists a need for studies that investigate communities of co-distributed anuran species representing a variety of host ecologies across complex environmental landscapes to better understand, and predict, expected future shifts in anuran skin microbiomes as climatic and habitat changes progress.

**Figure 1:**
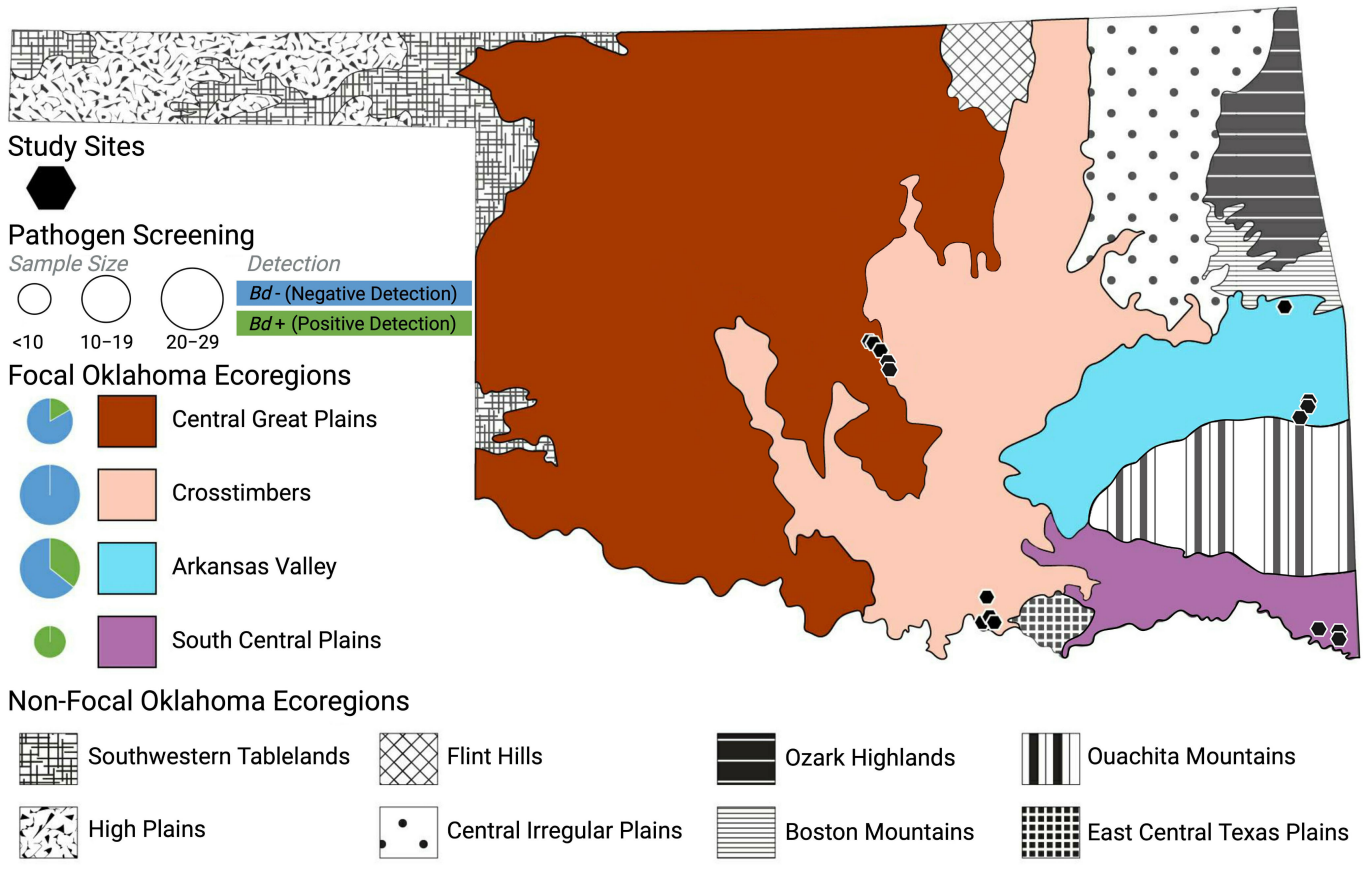
Map of Oklahoma depicting the 12 distinct ecoregions found in the state. The four focal ecoregions are distinguished based on color: burnt red/orange = Central Great Plains, peach = Crosstimbers, light blue = Arkansas Valley, purple = South Central Plains. All other (non-focal) ecoregions are defined by distinct patterns in grayscale. Sites surveyed for microbiome and disease samples are shown with black hexagons on the map. Pathogen screening results are summarized for each focal ecoregion with circular pie chart showing the percentage of screened samples positive (green) or negative (blue) for *Bd*. Each pie chart is size corrected to be proportional to sample size.

In this study, we evaluate the skin microbial communities of five widely distributed species of frogs across four major Oklahoma ecoregions to elucidate the roles that geographic location, host evolutionary history, host ecology, and pathogen presence play in anuran skin microbiome assembly. Ecoregions are areas of general ecosystem similarity characterized by abiotic and biotic variables such as climate, geology, soil, and vegetation (Omernik, 1995; Bryce et al., 1999; McMahon et al., 2001). Oklahoma is one of only four states in the United States that possesses more than 10 distinct ecoregions (U.S. Environmental Protection Agency, 2013; **Figure 1**), with 29 native species of frogs distributed across broad regions of this complex landscape (Sievert and Sievert, 2021). Furthermore, recent efforts to determine and monitor the distribution and prevalence of *Bd* infection among amphibian communities across the state has resulted in robust pathogen datasets that are now publicly available (Marhanka et al., 2017; Watters et al., 2018; Watters et al., 2019; Watters et al., 2021). Therefore, the state represents an ideal spatial framework for investigating the evolutionary and ecological processes involved in anuran skin microbiome assembly dynamics across changing environments (McMahon et al., 2001; Omernik, 2004) and in the presence of growing threats from emerging infectious diseases (Marhanka et al., 2017; Watters et al., 2018; Watters et al., 2019; Watters et al., 2021).

We perform both intra- and interpopulation comparisons of frog skin microbiomes across four ecoregions in Oklahoma for populations of Blanchard’s cricket frog (*Acris blanchardi*), the American green tree frog (*Hyla cinerea*), members of the morphologically indistinguishable gray treefrog species complex comprised of Gray’s (*H*. *chrysoscelis*) and Cope’s gray (*H*. *versicolor*) treefrogs (Jaslow and Vogt, 1977), which we refer to as *H*. *chrysoscelis*/*versicolor*, the American bullfrog (*Rana catesbeiana*), and the coastal plains leopard frog (*R*. *sphenocephala*) (**Figure 1**; **Supplementary Table 1**). Through these comparisons, our study aims to address the roles that (1) geographic location, (2) host ecology, (3) host evolutionary history, and (4) pathogen presence play in anuran skin microbiome assemblage. If geographic location has a dominant role in shaping frog skin microbial communities, we expect regional populations of each host species to possess distinct skin microbiomes. Conversely, if skin microbiome assemblage is impacted largely by host ecology, we expect frog species with similar life histories (aquatic, semi-aquatic, arboreal) to possess similar microbial community structure and diversity, regardless of their evolutionary history or geographic location. Furthermore, by comparing populations of closely related taxa within two distinct anuran families, we test whether host evolutionary history plays a role in microbiome assemblage by examining whether closely related species harbor greater microbial similarities than can be explained by geographic location or host ecology. Finally, if pathogen presence drives differences in skin microbiome diversity, we predict that anurans infected with *Bd* will possess distinct skin microbiomes when compared to frogs that tested negative for the fungus. Results from this comparative study contribute to our understanding of the environmental and host-associated processes that drive vertebrate skin microbiome assemblage. Furthermore, we provide the first assessment of variation in skin microbiomes among frog species spanning an ecological gradient in North America, allowing for future studies to investigate the impact environmental stressors, disease, and urbanization will have on population and species persistence.

## Materials and methods

### Sample collection

Fieldwork for this study was conducted from 2015–2021 (excluding 2020 due to the onset of the global pandemic) across four distinct ecoregions in Oklahoma: Arkansas Valley, Central Great Plains, Crosstimbers, and South Central Plains (**Figure 1**). These data were collected during a multi-year effort to sample amphibian microbiomes statewide. Frog communities from each ecoregion were sampled across a minimum of two years, while others were sampled over numerous years (**Supplementary Table 1**). These four focal ecoregions can be distinguished based on their geology, vegetation, and general location within the state, with the Arkansas Valley and South Central Plains ecoregions located in the eastern part of the state, and the Central Great Plains and Crosstimbers ecoregions spanning the central portion of Oklahoma (**Figure 1**; Woods et al., 2005). The Arkansas Valley is home to the richest fish fauna in Oklahoma and its landscape includes plains, hills, floodplains, wooded areas, and scattered mountains (Woods et al., 2005). The Central Great Plains ecoregion is characterized by red sedimentary rocks, scattered hills, salt plains, low mountains, and sandy flats (Woods et al., 2005). The natural vegetation of this ecoregion is mostly mixed grass prairie; therefore, there is almost no forest coverage and much of the land is used for rangeland, cropland, and oil extraction. A mixture of woodland, savanna, and prairie characterize the Crosstimbers ecoregion which serves as the border between the moister, more forested eastern ecoregions from the more arid, prairie-dominated western areas within the state (Woods et al., 2005). Finally, the humid South Central Plains ecoregion is comprised of floodplains, wetlands, forests, savannas, and some pastureland (Woods et al., 2005).

Four of our focal host species (*Acris blanchardi*, *Hyla chrysoscelis*/*versicolor*, *Rana catesbeiana*, and *R*. *sphenocephala*) are distributed across all four ecoregions, excluding *H*. *cinerea*, which has a range that spans the southeastern portion of the state only (Sievert and Sievert, 2021). Conversely, the range of *R*. *catesbeiana* encompasses the entire state and *A*. *blanchardi* is found in all portions of the state, excluding the northwestern “panhandle” of Oklahoma. Lastly, *Hyla chrysoscelis*/*versicolor* and *R*. *sphenocephala* are found only in the central and eastern parts of the state (Sievert and Sievert, 2021). Based on these distributions, we sampled the skin microbial communities of *Rana catesbeiana* and *R*. *sphenocephala* individuals from the Central Great Plains and South Central Plains ecoregions and *A*. *blanchardi*, *Hyla cinerea*, and *H*. *chrysoscelis*/*versicolor* populations in the Arkansas Valley and Crosstimbers ecoregions (**Figure 1**). These five species can also be distinguished based on their evolutionary histories, as *A*. *blanchardi*, *H. cinerea*, and *H*. *chrysoscelis*/*versicolor* belong to the family Hylidae (superfamily Hyloidea), whereas both *Rana* species are members of the Ranidae family (superfamily Ranoidea; Hime et al., 2021). Additionally, the focal species have different ecological preferences. For example, *A*. *blanchardi* and *R*. *sphenocephala* are semi-aquatic and found most often on the banks of waterbodies (Lehtinen and Skinner, 2006; Meade, 2008), while *R*. *catesbeiana* is fully aquatic and these frogs spend most of their time submerged in the shallow portions of ponds, lakes, swamps, and streams (Bruening, 2002). In contrast, both *Hyla* species prefer arboreal habitats (Martof et al., 1980; Harding, 1997); however, *H*. *cinerea* often rests on the shorter green vegetation that surrounds the banks of waterbodies. Conversely, *H*. *chrysoscelis*/*versicolor* is most often found higher in trees that are sometimes further away from water sources (Sievert and Sievert, 2021).

Skin microbiome samples were collected by rubbing a large, sterile rayon swab (Puritan Medical Products, Guilford, ME, USA) across the epidermis of an individual’s back, stomach, legs, and the webbing of both feet five times each (each down and back stroke was considered a single pass). We swabbed 179 individuals representing the five focal species: *Acris blanchardi* (N = 38), *Hyla chrysoscelis*/*versicolor* (N = 37), *H*. *cinerea* (N = 37), *Rana catesbeiana* (N = 42), and *R*. *sphenocephala* (N = 25; **Supplementary Table 1**). All swabs were preserved in liquid nitrogen or placed on ice immediately after collection until they were transferred to a -20°C freezer. Samples stored on ice temporarily were transferred to a -20°C freezer within 2–4 hours of collection. All samples were collected in strict accordance with the regulations established by the University of Oklahoma’s Institutional Animal Care and Use Committee (IACUC Permit Nos: R17-031, R21-005, T15-002, and T21-001). Each author secured Scientific Collectors Permits annually through the Oklahoma Department of Wildlife Conservation (ODWC).

### DNA extraction, polymerase chain reaction (PCR) amplification and sequencing

Genomic DNA was extracted from all 179 swabs using ZymoBIOMICS DNA Miniprep kits (Zymo Research Products, Irvine, CA, USA) in the Shared Genomics Core facilities of the Sam Noble Museum. The DNA concentration for a random subset of 50 extracted samples was determined using a Qubit 4 Fluorometer (Thermofisher Scientific, Waltham, Massachusetts, USA). Ten negative controls (i.e., ZymoBIOMICS reagents only, without a swab sample) were extracted alongside the 179 focal samples, nine of which were amplified and sequenced. Additionally, 75 μL of the ZymoBIOMICS Microbial Community Standard (i.e., a mock microbial community of known concentration; Zymo Research Products, Irvine, CA, USA) was extracted with the skin microbiome samples. We amplified and sequenced the 179 focal skin microbiome samples, nine extraction negative controls, two sets of ZymoBIOMICS Microbial Community Standards (Cat. No. D6300 and D6305, Zymo Research Products, Irvine, CA, USA), and two PCR-negative controls (one on each 96-well plate) using the methods outlined in Smith et al. (2021) and explained here in brief. We amplified the V4 hypervariable region of the 16S rRNA gene using primers and adapter sequences described in Kozich et al. (2013). After gel electrophoresis and bead clean-up (KAPA Pure Beads; Roche Sequencing Solutions, Pleasanton, CA, USA), we quantified each sample and normalized to 6 nM of DNA before pooling the samples into a single sterile, 1.5 mL microcentrifuge tube. Sequencing was performed at the University of Oklahoma Consolidated Core Lab using the 2x250 bp paired-end sequencing on a single run of an Illumina MiSeq.

### Sequence analysis

Adapter sequences described in Kozich et al. (2013) were trimmed from the paired-end raw sequencing reads using AdapterRemoval v2 and the following parameters—minquality: 30, trimqualities, maxns: 0, trimns, threads: 18 (Schubert et al., 2016). The sequence data was then imported into QIIME 2 (Bolyen et al., 2019) where *de novo* chimera checking and removal was performed using VSEARCH (Rognes et al., 2016) and UCHIME (Edgar et al., 2011). Then, the nonchimeric sequences were clustered into operational taxonomic units (OTUs) with a closed-reference OTU database at 97% sequencing similarity using VSEARCH (Rognes et al., 2016) against the Silva 138 database (Quast et al., 2012). Among all samples, including positive and negative controls, 4,050,580 sequences were clustered into 22,641 OTUs. Once we removed the positive and negative controls for downstream analyses, 20,542 OTUs and 3,469,844 sequences remained. We rarefied the OTU table to a sequencing depth of 1,000 for analysis based on our specific dataset and the associated rarefaction curves (**Supplementary Figure 1**), which removed 66 samples (**Supplementary Table 2**). These 66 samples were excluded from analysis due to low sequence counts when compared to the other samples (N = 113; **Supplementary Table 2**) which amplified well above a sequence count of 1,000. Raw sequence data generated in this study can be found in the Sequence Read Archive (SRA) under BioProject PRJNA1024480.

### Statistical analysis

To test how host species, family, ecology, and ecoregion correlated with differences in frog skin microbial communities, alpha diversity (within groups) and beta diversity (between groups) analyses were performed using the QIIME 2 software package. Diversity analyses were also performed on each species separately to determine if skin microbiomes differed among host communities occupying distinct ecoregions within the state, and a p*-*value or q-value < 0.05 was considered a statistically significant difference. Alpha and beta diversity comparisons were conducted using the alpha-group-significance and beta-group-significance plugins in QIIME 2, which performs Kruskal-Wallis tests (alpha diversity) and Pairwise PERMANOVAs (beta diversity) to test group significance (Anderson, 2001). When multiple pairwise comparisons were conducted, we reported q-values instead of p-values to correct for multiple tests (Storey, 2002; Storey, 2003; Storey and Tibshirani, 2003). Alpha diversity analyses were performed on three metrics of diversity Shannon Diversity, Faith’s Phylogenetic Diversity, and Observed OTUs using QIIME 2 (Bolyen et al., 2019). To evaluate how the skin microbiomes differed based on host species, family, and ecology (aquatic, semi-aquatic, arboreal), we analyzed beta diversity of skin samples using the phylogeny-based distance matrices Unweighted- and Weighted-Unifrac (Lozupone et al., 2007; Lozupone et al., 2011). Additionally, we performed these two analyses on the data from each focal frog species separately to determine if skin microbiomes differed between individuals of a single species occupying two distinct ecoregions. All beta diversity analyses were visualized by principal coordinate analysis (PCoA) using Qiime 2R (Bisanz, 2018) and Tidyverse (Wickham, 2017) packages in R version 4.2.1 (R Core Team, 2022). To understand the impact that host evolutionary history had on frog skin microbial diversity, we calculated phylogenetic signal using the three alpha diversity estimates (Shannon Diversity, Faith’s Phylogenetic Diversity, and Observed OTUs) and the R packages ape (Paradis and Schliep, 2019) and brms (Bürkner, 2017). The host phylogeny was obtained through VertLife (Jetz and Pyron, 2018). Lastly, we utilized the package PARTY in R (Hothorn et al., 2006; R Core Team, 2022) to perform Classification and Regression Tree (CART) analyses on a subset of skin microbiome samples that also had *Bd* disease results for the host organisms (N = 71) to determine which variable (host species, host family, ecoregion, ecology, infection status, or infection load) accounted for most of the variance observed in each of our three alpha diversity metrics.

## Results

Among the 179 skin microbiome swabs we collected from our five focal frog species, we obtained 4,050,580 sequences which were clustered into 22,641 OTUs using a 97% sequence similarity threshold against the Silva database (version 138, Quast et al., 2012). After rarefying the 179 samples to a sequencing depth of 1,000 sequences and removing positive and negative control samples, 113 samples and 7,694 OTUs remained and were used in subsequent analyses (*A*. *blanchardi* [N = 17], *H*. *chrysoscelis*/*versicolor* [N = 27], *H*. *cinerea* [N = 24], *R*. *catesbeiana* [N = 30], and *R*. *sphenocephala* [N = 15]; **Supplementary Table 2**). Visualization of the data obtained from the two positive control samples indicated that the community compositions of the Extraction-Stage and PCR-Stage ZymoBIOMICS Microbial Community Standards were similar, suggesting that our extraction-to-analysis workflow performed consistently (**Supplementary Figure 2**).

### Taxonomic composition of skin microbial communities across host species, ecoregion, and ecology

**Figure 2:**
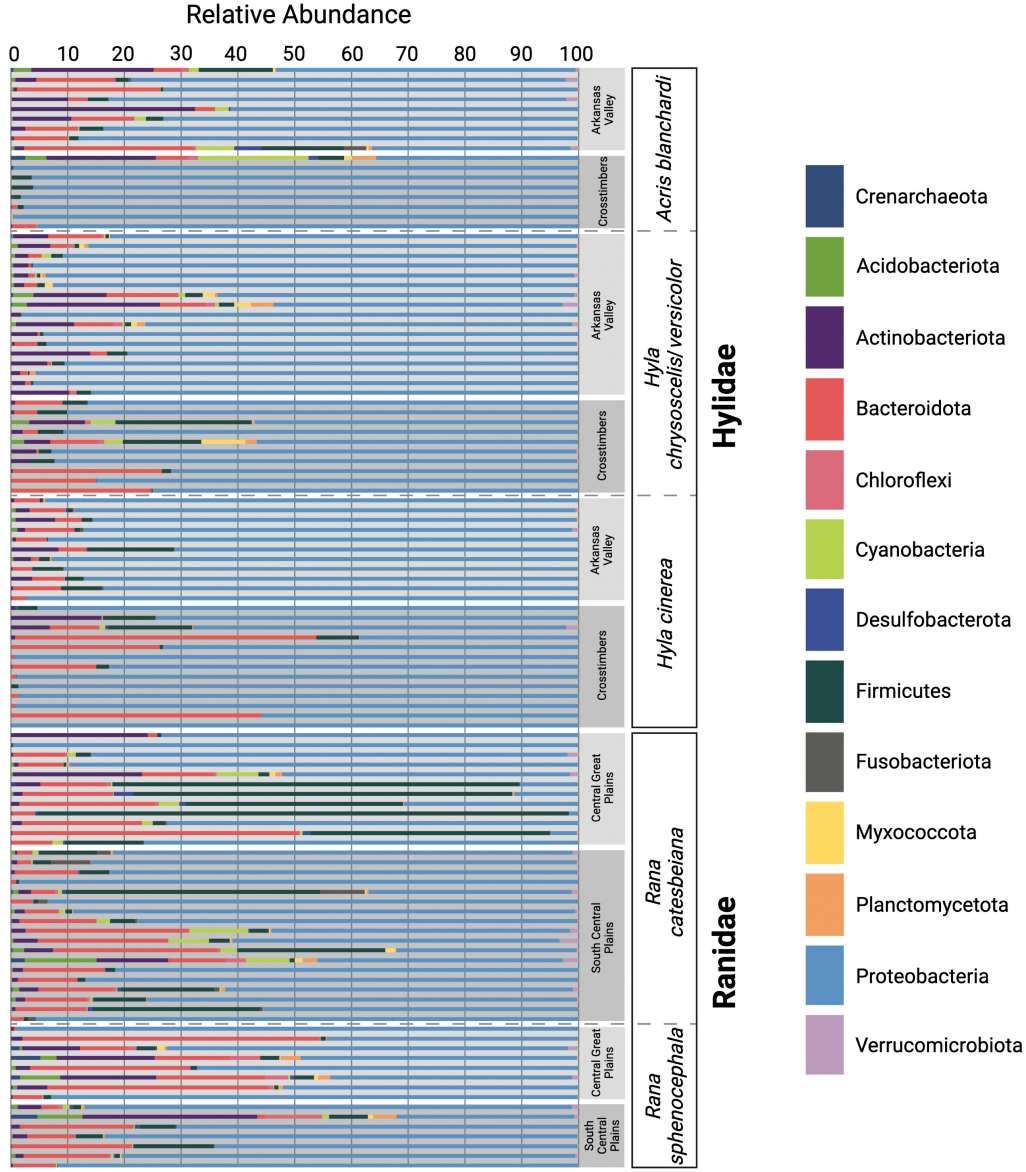
Relative abundances of the dominant microbial phyla acquired through 16S rRNA amplicon sequencing. Each horizontal bar represents an individual swab. Samples are grouped by host family, host species, and ecoregion.

Proteobacteria was the most abundant bacterial phylum found among the 113 skin microbiome samples (relative abundance of 75.48%; **Figure 2**). Three other dominant phyla (average relative abundance > 1.0% excluding values equal to zero; Suenami et al., 2019) were found in the majority of the samples (N = 111)—Bacteroidetes (9.71%), Firmicutes (6.82%), and Actinobacteria (4.30%; **Figure 2**). Among all aquatic (*R*. *catesbeiana*) samples, the most dominant phyla was Proteobacteria (62.67%) followed by Firmicutes (16.49%), Bacteroidetes (12.11%), Actinobacteria (3.23%), Cyanobacteria (1.98%), Fusobacteria (1.36%), and Acidobacteria (1.07%; **Figure 2**). Chloroflexi (3.54%; N = 1), Desulfobacterota (1.25%–3.45%; N = 3), Gemmatimonadota (1.84%; N = 1), Myxococcota (1.34%–1.82%; N = 2), Planctomycetota (1.20%–2.55%; N = 2), and Verrucomicrobia (1.09%–3.40%; N = 8) were found in varying abundances among *R*. *catesbeiana* skin samples (**Figure 2**).

The most dominant phylum among semi-aquatic (*A*. *blanchardi* and *R*. *sphenocephala*) samples was Proteobacteria with an average relative abundance of 73.60%. Bacteroidetes was the second most abundant (11.70%) followed by Actinobacteria (6.29%), then Firmicutes (3.27%; **Figure 2**). Additionally, Acidobacteria (1.07%–7.54%; N = 6), Chloroflexi (1.35%–9.57%; N = 6), Cyanobacteria (1.15%–18.65%; N = 7), Myxococcota (1.32%–1.37%; N = 2), Planctomycetes (2.07%–4.11%; N = 4), and Verrucomicrobia (1.19%–2.35%; N = 6) were found among samples from both semi-aquatic species (**Figure 2**). Interestingly, the Archaea phylum Crenarchaeota was present within four *R*. *sphenocephala* samples (1.51%–5.07%) and one *A*. *blanchardi* sample (2.4%; **Figure 2**). With the exception of one *R*. *catesbeiana* sample (2.36%), Crenarchaeota was not found in abundances greater than 1% in any of the other samples. Additionally, four phyla, Actinobacteria, Bacteroidetes, Cyanobacteria, and Verrucomicrobia were present in abundances greater than 1% (1.64%–32.38%, 3.48%–29.52%, 1.70%–6.65%, and 1.43%–2.35%, respectively) among *A*. *blanchardi* skin samples from the Arkansas Valley only with the exception of two samples from the Crosstimbers ecoregion that had relative abundances greater than 1% for these four phyla (**Figure 2**). Conversely, all but one of the Crosstimbers *A*. *blanchardi* skin samples were dominated by Proteobacteria (95.40%–99.66%; **Figure 2**). Lastly, two phyla, Desulfobacteria and Fusobacteria were found among samples from *A*. *blanchardi* and *R*. *catesbeiana* only, in abundances ranging from 1.25%–4.66% (Desulfobacteria) and 1.35%–7.80% (Fusobacteria).

Among skin samples collected from the two arboreal frog species (*H*. *chrysoscelis*/*versicolor* and *H*. *cinerea*), the same four dominant phyla were found—Proteobacteria (84.2%), Bacteroidetes (7.03%), Actinobacteria (3.70%), and Firmicutes (3.22%; **Figure 2**). However, the phyla Acidobacteriota, Chloroflexi, Cyanobacteria, Myxococcota, and Planctomycetes were represented in abundances greater than 1% (1.05%–7.5%) in 4–5 *H*. *chrysoscelis*/*versicolor* samples only with the exception of Acidobacteriota which had a relative abundance of 1.07% in a single sample of *H*. *cinerea* (**Figure 2**).

### Analyses of mechanistic processes influencing host microbial diversity

We utilized several alpha diversity metrics to test for significant differences among the skin microbiome samples using the Kruskal-Wallis test. First, Faith’s Phylogenetic Diversity analysis found significant differences among *A*. *blanchardi* and both *Rana* species (*A*. *blanchardi* vs. *R*. *catesbeiana*: H = 5.61; q-value = 0.03; *A*. *blanchardi* vs. *R*. *sphenocephala*: H = 7.50; q-value = 0.016; **Supplementary Figure 3**). Additionally, this analysis found that the skin microbial diversity of both *Hyla* species were significantly different from both *Rana* species (*H*. *chrysoscelis*/*versicolor* vs. *R*. *catesbeiana*: H = 7.04; q-value = 0.016; *H*. *chrysoscelis*/*versicolor* vs. *R*. *sphenocephala*: H = 7.10; q-value = 0.016; *H*. *cinerea* vs. *R*. *catesbeiana*: H = 18.79; q-value = 0.0001; *H*. *cinerea* vs. *R*. *sphenocephala*: H = 17.04; q-value = 0.0002; **Supplementary Figure 3**). However, no significant differences were found when comparing the skin microbiomes of the three species within the Hylidae family (*A*. *blanchardi* vs. *H*. *chrysoscelis*/*versicolor*: H = 0.106; q-value = 0.745; *A*. *blanchardi* vs. *H*. *cinerea*: H = 1.89; q-value = 0.211; *H*. *chrysoscelis*/*versicolor* vs. *H*. *cinerea*: H = 3.28; q-value = 0.100; **Supplementary Figure 3**). Additionally, we found no significant difference when comparing the skin microbiomes of the two Ranidae species (*R*. *catesbeiana* vs. *R*. *sphenocephala*: H = 1.17; q-value = 0.31; **Supplementary Figure 3**). In comparison, the Observed OTU analysis indicated significant differences between *R*. *sphenocephala* and both *Hyla* species only (*H*. *chrysoscelis*/*versicolor*: H = 8.88; q-value = 0.014; *H*. *cinerea*: H = 12.82; q-value = 0.003; **Supplementary Figure 3**). Results from the Shannon Diversity analysis yielded significant differences between *A*. *blanchardi* and *H*. *cinerea* skin microbiome samples (H = 5.67; q-value = 0.04; **Supplementary Figure 3**). Additionally, the skin microbial communities of both *Hyla* species were significantly different from the two *Rana* species with the exception of *H*. *chrysoscelis*/*versicolor* vs. R. *catesbeiana*: H = 4.79; q-value = 0.057 (*H*. *chrysoscelis*/*versicolor* vs. *R*. *sphenocephala*: H = 7.52; q-value = 0.03; *H*. *cinerea* vs. *R*. *catesbeiana*: H = 6.82; q-value = 0.03; *H*. *cinerea* vs. *R*. *sphenocephala*: H = 9.19; q-value = 0.024; **Supplementary Figure 3**).

Subsequently, we incorporated the alpha diversity data into two additional analyses: (1) a Phylogenetic Generalized Linear Mixed Model to test for phylogenetic signal, and (2) a CART analysis to determine which variable (host species, host family, ecoregion, ecology, infection status, or infection load) accounted for most of the variation within skin microbial communities associated with the subset of frogs that had corresponding *Bd* disease data (N = 71). We did not detect phylogenetic signal among the skin microbiomes of our five focal host species using the three alpha diversity metrics: Shannon (*h*^2^ = 0.22, Estimated Error = 0.19), Faith’s Phylogenetic Diversity (*h*^2^ = 0.22, Estimated Error = 0.18), and Observed OTUs (*h*^2^ = 0.18, Estimated Error = 0.16). Additionally, the results of the CART analysis indicated that, when analyzing Shannon Diversity, ecology was the only variable (χ2 = 16.626; p-value = 0.006) that explained the variance in skin microbiome diversity, with aquatic and semi-aquatic frogs having significantly higher Shannon Diversity when compared to arboreal frogs (**Supplementary Figure 4**). The Observed OTU CART analysis found that ecoregion explained the majority of the variance, with samples from the Crosstimbers ecoregion having significantly fewer OTUs when compared to samples from the Arkansas Valley, Central Great Plains, and South Central Plains ecoregions (χ2 = 21.852, p-value < 0.001; **Supplementary Figure 5**). Among samples from the Arkansas Valley, Central Great Plains, and South Central Plains ecoregions, we found that ecology explained the remaining variance in skin microbiome diversity, with samples from semi-aquatic frogs having more OTUs compared to aquatic and arboreal frogs (χ2 = 18.951, p-value = 0.002; **Supplementary Figure 5**). Lastly, results from the CART analysis with Faith’s Phylogenetic Diversity indicated that ecoregion explained all of the variance within our focal skin microbiome samples with samples from the Crosstimbers ecoregion being significantly less diverse than samples from the other three tested ecoregions (χ2 = 27.449, p-value < 0.001; **Supplementary Figure 6**). Additionally, samples from the Arkansas Valley, Central Great Plains, and South Central Plains ecoregions were further split, with samples from the Central Great Plains and South Central Plains being more diverse than samples from the Arkansas Valley ecoregion (χ2 = 13.936, p-value = 0.012; **Supplementary Figure 6**). As such, results from our CART analysis suggest that host ecology and geographic location are the primary variables that explain the variance in our focal skin microbiome samples. In contrast, host species, host family, *Bd* status, and *Bd* infection intensity did not explain any of the variance in our microbiome samples.

**Figure 3:**
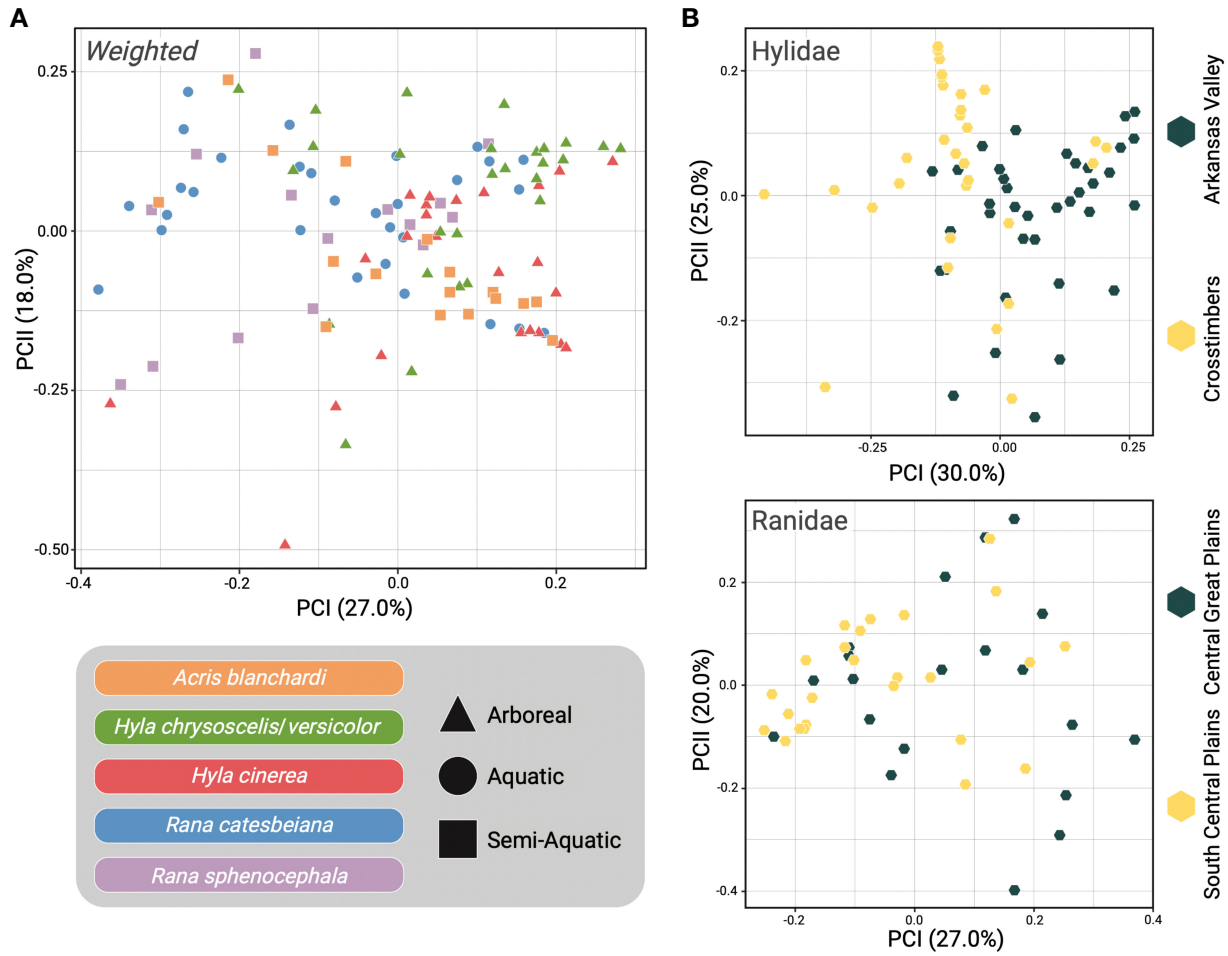
Beta diversity comparisons using Weighted-Unifrac distance. **(A)** Principal Coordinates Analysis (PCoA) of all samples with host species represented by distinct colors and point shape indicating if the sample was from an arboreal (triangle), aquatic (circle), or semi-aquatic (square) frog. **(B)** Samples were separated into their respective host families with Hylidae samples plotted in the PCoA at the top and Ranidae samples on the bottom. Point color indicates the ecoregion that the sample was collected from (yellow = Crosstimbers and South Central Plains, gray = Arkansas Valley and Central Great Plains).

Unweighted- and Weighted-Unifrac distance matrices were used to analyze beta diversity among skin microbiome samples. Unifrac is a phylogenetic distance metric that measures the difference between microbial communities based on the degree of divergence between different sequences (Lozupone and Knight, 2005). There are two types of Unifrac distances, Unweighted and Weighted, with Weighted-Unifrac also accounting for differences in the relative abundances of microbial taxa within samples (Lozupone et al., 2007). We report findings from both tests here as they produce different but complementary findings (**Figure 3**; **Supplementary Figure 7**). In summary, both analyses indicated that there were significant differences in the skin microbiomes of the focal host species, with the exception of *A*. *blanchardi* vs. both *Hyla* species (Unweighted) and *A*. *blanchardi* vs. *H*. *cinerea* (Weighted; **Supplementary Materials**). Additionally, both analyses found significant differences between host families and host ecologies (**Figure 3**; **Supplementary Figure 7**). Further, intra-specific comparisons of skin microbiomes between ecoregions yielded significant differences within most host species (with the exception of *H*. *chrysoscelis*/*versicolor* and *R*. *sphenocephala*; **Supplementary Materials**). For more detailed comparisons, please refer to the **Supplementary Materials**.

## Discussion

Our study sampled the skin microbial communities of anuran hosts across distinct ecoregions in Oklahoma to determine if geographic location, host ecology, pathogen infection, or host evolutionary history correlated with differences in frog skin microbiomes. Results from the CART analysis indicated that host ecology and geographic location were the primary drivers of the skin microbiome diversity differences we observed among the five focal frog species sampled (**Supplementary Figures 4–6**). Although we observed some compositional differences among skin microbiome samples based on the geographic locations of our hosts, such patterns were not shared across all five host species (**Figure 2**). Interestingly, the most prominent differences were observed among *A*. *blanchardi* skin microbiomes, which differed profoundly between individuals from the two unique ecoregions we sampled (**Figure 2**). Four phyla, Actinobacteria, Bacteroidetes, Cyanobacteria, and Verrucomicrobia, were present in abundances greater than 1% among samples from Arkansas Valley only, with the exception of two samples from the Crosstimbers ecoregion. This is likely because most of the *A*. *blanchardi* samples from the Crosstimbers were dominated largely by Proteobacteria (**Figure 2**). For the geographic location hypothesis to be fully supported, we would expect to see more ecoregion-specific compositional differences among the skin microbiomes of our other focal anuran species. Additionally, *Bd* infection status and severity did not emerge as primary drivers of anuran skin microbiome diversity among our focal samples (**Supplementary Figures 4–6**). However, previous studies have found that *Bd* infection impacts the composition of anuran skin microbiomes (Rebollar et al., 2016; Familiar-López et al., 2017; Rebollar and Harris, 2019; Jani et al., 2021), and disease data was only available for a subset of samples in our study (N = 71 out of 113 samples). Further, while both *Hyla* species had associated disease data for 8–13 individuals per ecoregion, *R*. *catesbeiana* and *R*. *sphenocephala* only had disease information for 2–6 individuals per ecoregion, and no *R*. *catesbeiana* individuals from the South Central Plains ecoregion had corresponding *Bd* disease data (**Figure 1**; **Supplementary Table 1**). As such, it may be difficult to detect a correlation between *Bd* disease status/severity and microbiome compositional differences when sample sizes are small. Therefore, increased sampling is needed to further investigate how *Bd* disease status and severity alters anuran skin microbiome diversity. We did not find evidence of phylogenetic signal among the skin samples from our focal hosts, indicating that anuran skin microbiomes are influenced by host ecology and geographic location, whereas host evolutionary history and pathogen infection did not emerge as primary drivers.

Four dominant bacterial phyla emerged within nearly all samples (N = 111/113), Actinobacteria, Bacteroidetes, Firmicutes, and Proteobacteria, a pattern consistent with findings from other amphibian skin microbiome studies to date (Kueneman et al., 2014; Walke et al., 2014; Walke and Belden, 2016; Bletz et al., 2017). Additionally, previous research has indicated that Chloroflexi (Walke et al., 2014; Ellison et al., 2019), Cyanobacteria (McKenzie et al., 2012; Walke et al., 2014; Ellison et al., 2019), and Planctomycetes (Jiménez and Sommer, 2017; Prado-Irwin et al., 2017; Muletz Wolz et al., 2018; Albecker et al., 2019; Ellison et al., 2019) are also dominant members of the amphibian skin microbiome. Therefore, it is not surprising that we found these phyla in abundances greater than 1% among samples from four of our focal host anurans (excluding *H*. *cinerea*). Although Firmicutes was a dominant phylum among skin microbiome samples from all of our focal host species, some *R*. *catesbeiana* samples had abundances of Firmicutes as high as 93.54% (**Figure 2**). This was surprising given that previous research, and results from our study, indicate that Proteobacteria dominates the amphibian skin microbiome (Walke et al., 2014; Walke et al., 2015; Bataille et al., 2016; Medina et al., 2017; Muletz Wolz et al., 2018; **Figure 2**). However, studies have also found that skin microbial communities dominated by Firmicutes are associated with the presence of skin lesions caused by psoriasis (Gao et al., 2008; Yan et al., 2017) and atopic dermatitis (Zheng et al., 2019) in humans, and papillomatous digital dermatitis in cows (Santos et al., 2012). Therefore, it is possible that the *R*. *catesbeiana* individuals with high abundances of Firmicutes in their microbiomes could have experienced prior skin infections or were infected at the time of sampling. However, only a small subset (N = 6) of the focal *R*. *catesbeiana* hosts had associated *Bd* infection data. As such, we cannot confirm if *Bd* presence is correlated with an abundance of Firmicutes in the skin microbiomes of this species at this time, but future studies should investigate if amphibian skin lesions are also colonized by Firmicutes, as observed in the mammalian skin microbiome literature (Santos et al., 2012; Zheng et al., 2019).

We found species-specific compositional differences among the five anuran species sampled. Notably, the Archaea phylum Crenarchaeota was found among aquatic and semi-aquatic species only (*A*. *blanchardi*, *R*. *catesbeiana*, and *R*. *sphenocephala*; **Figure 2**). All cultured Crenarchaeota are extreme thermophiles (Ochsenreiter et al., 2003); however, with increased use of culture-independent sequencing methods, studies have indicated that there are members of the Crenarchaeota phylum that are associated with freshwater sediments and other soil environments (Ochsenreiter et al., 2003; Brochier-Armanet et al., 2008). *Acris blanchardi*, *R*. *catesbeiana*, and *R*. *sphenocephala* are commonly encountered within or around water and marshy habitats, which could explain why Crenarchaeota was found on the skin of these species and not the two arboreal host species (*H*. *cinerea* and H. *chrysoscelis*/*versicolor*). Additionally, we found Myxococcota among skin microbiome samples from four of our host species (excluding *H*. *cinerea*). Members of this phylum colonize soil and rotting plant material, including decaying wood and tree bark, but also freshwater environments (Reichenbach, 1999). *Hyla cinerea* is an arboreal frog which is found most often on green, weedy vegetation on the margin of swamps and ponds (Sievert and Sievert, 2021), possibly limiting their exposure to bacteria that inhabit soil and decaying plant material, such as Myxococcota. In contrast, *H*. *chrysoscelis*/*versicolor* inhabits woody vegetation such as shrubs and trees (Sievert and Sievert, 2021), which could explain why Myxococcota was found on the skin of *H*. *chrysoscelis*/*versicolor* and not *H*. *cinerea*.

After a recent splitting of the bacterial class Deltaproteobacteria, Myxococcota and Desulfobacterota emerged as two distinct phyla (Waite et al., 2020; Langwig et al., 2022). Desulfobacterota was detected among a few *A*. *blandchardi* and *R*. *catesbeiana* samples. This phylum has been found in Oklahoma wetland sediment previously (Murphy et al., 2021), which may be why we detected members of this phylum on the skin of *A*. *blandchardi* and *R*. *catesbeiana*, two host species that are most often found within or around waterbodies, and emphasizes the importance of collecting environmental samples alongside host microbiome samples. To our knowledge, no studies have reported Crenarchaeota, Desulfobacterota, or Myxococcota as dominant members of the amphibian skin microbiome. However, the recent reclassification of Deltaproteobacteria could explain why Desulfobacterota and Myxococcota have not yet been detected on the skin of amphibians. Deltaproteobacteria was found on the skin of frogs from Madagascar in small relative abundances (1.6%; Bletz et al., 2017), and among the different environmental samples collected by Bletz et al. (2017). Therefore, more studies are needed to determine if Crenarchaeota, Desulfobacterota, and Myxococcota are prominent members of the amphibian skin microbiome or if our findings are an example of host frogs obtaining some of their skin microbiota from their surrounding environment, a concept which has been discussed in numerous other studies (Belden and Harris, 2007; Becker et al., 2014; Loudon et al., 2014b; Bletz et al., 2017; Jiménez and Sommer, 2017; Kueneman et al., 2019; Rebollar and Harris, 2019; Hernández-Gómez et al., 2020; Barnes et al., 2021). For example, soil and water can act as reservoirs for microbiota (Loudon et al., 2014b) which can then colonize the skin of amphibians (Belden and Harris, 2007; Becker et al., 2014; Hernández-Gómez et al., 2020; Barnes et al., 2021). It is thought that these reservoirs support the presence of rare bacterial taxa within the amphibian skin microbiome (Loudon et al., 2014b) and changes to microbial communities in the environment may impact amphibian skin microbiome composition and diversity (Belden and Harris, 2007). Given that the skin microbiome is critical for promoting amphibian immune system function and pathogen defense (Bernardo-Cravo et al., 2020; Rebollar et al., 2020), altering reservoir microbiota with captive breeding or head-start initiatives could impact host health (Belden and Harris, 2007; Becker et al., 2014; Loudon et al., 2014b). As such, future investigations which explore the exchange of rare microbes between the environmental and amphibian skin microbiomes are essential for understanding this complex dynamic and may have critical implications for conservation initiatives. To accomplish this, future studies should consider sampling, and subsequently sequencing, the environmental microbiome present at the exact location where the host organism is collected, a method which has been employed in several other studies to date (Walke et al., 2014; Bletz et al., 2017; Jiménez and Sommer, 2017). Implementing this methodology in future studies may improve our understanding of the mechanisms shaping the composition and diversity of anuran skin microbiomes while also providing a way to differentiate skin microbiome samples that have been potentially contaminated with environmental microbes from those collected from host organisms that have rare microbes within their skin microbiomes. Previous findings indicate that the same dominant microbial phyla comprise both environmental and amphibian skin microbiome samples (e.g., Acidobacteria, Actinobacteria, Bacteroidetes, Firmicutes, Proteobacteria) but in different relative abundances (Walke et al., 2014; Bletz et al., 2017; Hernández-Gómez et al., 2020; Barnes et al., 2021). Therefore, analyzing environmental microbiome samples alongside skin microbiome samples may help identify samples that have been contaminated with environmental microbiota because the compositions of the contaminated samples will be more similar to the environmental samples than to the focal skin microbiome samples. In contrast, we would expect that samples collected from hosts with rare microbial taxa in their skin microbiomes would have compositions that are more similar to the other hosts when compared to the environmental samples but with the addition of the rare microbial taxa.

Another consideration for future work is to expand the taxonomic scope of amphibian skin microbiome studies, particularly in a state like Oklahoma which is home to an impressive diversity of amphibian species distributed across a diverse landscape. For the purposes of our study, we aimed to sample co-distributed anuran species that are found commonly in the wild to allow for sufficiently large sample sizes and more robust comparisons of skin microbiomes among taxa. However, continued field surveys across central and eastern Oklahoma would enable expanded comparisons, both taxonomically (across orders, families, genera, and species) and geographically (across more ecoregions) in Oklahoma (**Figure 1**). For example, Oklahoma is home to several species of true toads (family Bufonidae), narrow-mouthed toads (family Microhylidae), and spadefoot toads (family Scaphiopodidae), along with other members of the focal families Hylidae and Ranidae from this study that lacked sufficient sample sizes to include in our dataset, such as *Pseudacris clarkii*, *P*. *crucifer*, *P*. *fouquettei*, and *P*. *streckeri* in the family Hylidae and *Rana areolata*, *R*. *blairi*, *R*. *clamitans*, and *R*. *palustris* in the family Ranidae (Sievert and Sievert, 2021). There is also an incredible diversity of salamanders (order Caudata) distributed across the eastern portion of the state which presents exciting opportunities for future research to compare and contrast the findings of this study with the skin microbiome diversity of this divergent clade of amphibians. Further, given the variable nature in which we sampled frog communities from the four focal ecoregions—frogs from certain ecoregions were sampled across multiple years, other sampled in only two years—future research should investigate to what degree anuran skin microbiomes vary across seasons or years (Douglas et al., 2021).

Symbiotic microbial communities serve fundamental roles in a variety of processes that promote host health. As such, investigating the environmental and evolutionary mechanisms that drive the assemblage of frog skin microbiomes remains essential as we begin to form a more comprehensive understanding of the factors impacting anuran health, persistence, and immunity. Results from this study suggest that multiple factors influence the structure of anuran skin microbial communities; however, host ecology plays the largest role in the assembly of skin microbiome diversity among our sampled Oklahoma frog communities. Comparative studies of skin microbiomes among co-distributed anuran species spanning complex landscapes allow us to better understand, and predict, impending shifts in anuran skin microbial communities in response to continued climate and habitat changes. The investigation of amphibian skin microbiomes remains critical as the vertebrate group continues to face population declines imposed by disease, habitat loss, pollution, and climatic changes.

## Data availability statement

The datasets presented in this study can be found in online repositories. The names of the repository/repositories and accession number are: NCBI SRA; PRJNA1024480.

## Ethics statement

The animal study was approved by University of Oklahoma’s Institutional Animal Care and Use Committee. The study was conducted in accordance with the local legislation and institutional requirements.

## Author contributions

SS: Conceptualization, Data curation, Formal Analysis, Investigation, Methodology, Project administration, Validation, Visualization, Writing – original draft. JW: Data curation, Funding acquisition, Resources, Writing – review & editing. CS: Conceptualization, Funding acquisition, Resources, Supervision, Validation, Writing – review & editing.
