# Supplementary material for: Host ecology drives frog skin microbiome diversity across ecotone in South-Central North America
Source: Front Microbiomes. 2023 Nov 7;2:1286985. doi: 10.3389/frmbi.2023.1286985 (PMC12993627; doi:10.3389/frmbi.2023.1286985)
Supplement: Supplementary file 1 [file DataSheet_1.pdf]

Supplementary Material

Host Ecology Drives Frog Skin Microbiome Diversity Across Ecotone in South-Central North America

Sierra N. Smith*, Jessa L. Watters, and Cameron D. Siler

*** Correspondence:** Sierra N. Smith, Sam Noble Oklahoma Museum of Natural History and School of Biological Sciences, University of Oklahoma, 730 Van Vleet Oval, Room 314, Norman, Oklahoma 73019, USA, sierrasmith@ou.edu

Supplementary Data: Detailed Unweighted- and Weighted-Unifrac beta diversity results

Unweighted- and Weighted-Unifrac distance matrices were used to analyze beta diversity among skin microbiome samples. The Unweighted-Unifrac analysis indicated that the skin microbiomes of *A. blanchardi* were significantly different from both *Rana* species (*R. catesbeiana*: pseudo-F = 2.81; q-value = 0.003; *R. sphenocephala*: pseudo-F = 2.27; q-value = 0.006; Supplementary Figure 7) but were not significantly different from either *Hyla* species (*H. chrysoscelis*/*versicolor*: pseudo-F = 1.34; q-value = 0.094; *H. cinerea*: pseudo-F = 1.39; q-value = 0.105). Additionally, the skin microbial communities of *H. chrysoscelis*/*versicolor* were significantly different from *H. cinerea* (pseudo-F = 2.00; q-value = 0.014), *R. catesbeiana* (pseudo-F = 3.81; q-value = 0.003), and *R. sphenocephala* (pseudo-F = 2.40; q-value = 0.006). Also, results from this analysis indicated that *H. cinerea* had significantly different skin microbial diversity when compared to both *Rana* species (*R. catesbeiana*: pseudo-F = 5.10; q-value = 0.003; *R. sphenocephala*: pseudo-F = 4.30; q-value = 0.003) and both *Rana* species differed from one another (*R. catesbeiana* vs. *R. sphenocephala*: pseudo-F = 2.00; q-value = 0.006). Based on the species-to-species comparisons, it is not surprising that skin microbiomes differed by host family (Hylidae vs. Ranidae: pseudo-F = 5.94; p-value = 0.001). When

we grouped the data into ecoregions, we found that samples from each ecoregion were significantly different from one another (Arkansas Valley vs. Central Great Plains: pseudo-F = 2.28; q-value = 0.002, Crosstimbers: pseudo-F = 4.69; q-value = 0.002, and South Central Plains: pseudo-F = 3.67; q-value = 0.002; Central Great Plains vs. Crosstimbers: pseudo-F = 4.49; q-value = 0.002 and South Central Plains: pseudo-F = 2.08; q-value = 0.004; Crosstimbers vs. South Central Plains: pseudo-F = 8.45; q-value = 0.002). More specifically, within each host species, except *H. chrysoscelis/versicolor* (Arkansas Valley vs. Crosstimbers: pseudo-F = 1.56; p-value = 0.057), significant differences were found among skin microbiome samples collected from the same species located in different ecoregions (*A. blanchardi*: Arkansas Valley vs. Crosstimbers: pseudo-F = 3.87; p-value = 0.001; *H. cinerea*: Arkansas Valley vs. Crosstimbers: pseudo-F = 3.09; p-value = 0.003; *R. catesbeiana*: Central Great Plains vs. South Central Plains: pseudo-F = 2.30; p-value = 0.002; *R. sphenocephala*: Central Great Plains vs. South Central Plains: pseudo-F = 1.34; p-value = 0.048; Supplementary Figure 7). Lastly, when we grouped samples based on the ecology of the hosts, we found significant differences between each habitat type: aquatic vs. arboreal (pseudo-F = 5.34; q-value = 0.002), aquatic vs. semi-aquatic (pseudo-F = 2.45; q-value = 0.002), and arboreal vs. semi-aquatic (pseudo-F = 2.33; q-value = 0.003; Supplementary Figure 7).

Weighted-Unifrac analysis of the skin microbiome samples found significant differences among all host species we compared with the exception of *A. blanchardi* vs. *H. cinerea* (pseudo-F = 1.75; q-value = 0.111). Additionally, significant differences were found among samples from each host family (Hylidae vs. Ranidae: pseudo-F = 8.96; p-value = 0.001). Skin microbiomes differed among all species found at distinct ecoregions (Arkansas Valley vs. Central Great Plains: pseudo-F = 9.83; q-value = 0.002, Crosstimbers: pseudo-F = 9.74; q-value = 0.002, and South Central Plains: pseudo-F = 4.52; q-value = 0.002; Central Great Plains vs. Crosstimbers: pseudo-F = 7.59; q-value = 0.002 and South Central Plains: pseudo-F = 2.89; q-value = 0.009; Crosstimbers vs. South Central Plains:

pseudo-F = 8.12; q-value = 0.002). With the exception of *R. sphenocephala* (Central Great Plains vs. South Central Plains: pseudo-F = 1.98; p-value = 0.094), the skin microbial communities of each host species differed between ecoregions (*A. blanchardi*: Arkansas Valley vs. Crosstimbers: pseudo-F = 3.14; p-value = 0.011; *H. chrysoscelis*/*versicolor*: Arkansas Valley vs. Crosstimbers: pseudo-F = 5.79; p-value = 0.001; *H. cinerea*: Arkansas Valley vs. Crosstimbers: pseudo-F = 4.92; p-value = 0.001; R. *catesbeiana*: Central Great Plains vs. South Central Plains: pseudo-F = 2.29; p-value = 0.038; Figure 3). Additionally, skin microbiomes differed based on host ecology (aquatic vs. arboreal: pseudo-F = 8.49; q-value = 0.003; aquatic vs. semi-aquatic: pseudo-F = 2.64; q-value = 0.019; arboreal vs. semi-aquatic: pseudo-F = 4.79; q-value = 0.003; Figure 3).

Supplementary Figure Legends

Supplementary Figure 1. Alpha diversity rarefaction curves based on Shannon Diversity and Number of OTUs. Each curve is representative of a single swab. Based on these curves, we decided to rarefy all samples to a sequencing depth of 1,000 sequences.

Supplementary Figure 2. Compositional comparison of two positive control samples (Zymo Research Products, Irvine, CA, USA): the ZymoBIOMICS microbial community DNA standard (Cat. No. D6305, Zymo Research Products) and the ZymoBIOMICS microbial community standard (Cat. No. D6300, Zymo Research Products) alongside the expected composition of both standards. The microbial community standard was extracted alongside the focal skin microbiome samples whereas the microbial DNA standard contained pre-extracted DNA that was amplified and sequenced with the other focal samples. The compositions of both standards were compared to assess any bias or error in our extraction and amplification methods. We found that there were little compositional differences between the two standards; however, two microbial species that comprised a small portion of the expected composition, *Cryptococcus neoformans* and *Saccharomyces cerevisiae* were missing from the two positive control samples. This could indicate that our methodologies may have been less

sensitive to certain microbial taxa that comprised a small percentage of the overall microbiome composition within our samples.

Supplementary Figure 3. Alpha diversity (Faith's Phylogenetic Diversity, Top; Shannon Diversity, Middle; Observed OTUs, Bottom) comparisons of microbial OTUs by host species. The host species are represented by distinct colors as indicated in the figure legend. Figure was created with BioRender.com.

Supplementary Figure 4. Results of the Classification and Regression Tree analysis indicated that host ecology explained all of the variance in Shannon Diversity among our focal samples. Both aquatic and semi-aquatic frogs had significantly higher diversity than the arboreal species.

Supplementary Figure 5. Classification and Regression Tree analysis revealed that ecoregion explained most of the variance found in the number of Operational Taxonomic Units (i.e., Observed OTUs) within our focal samples. Samples from the Crosstimbers ecoregion had significantly less OTUs when compared to samples from the other three ecoregions. Additionally, among frogs from the Arkansas Valley, Central Great Plains, and South Central Plains ecoregions, ecology explained the remaining variance in the number of OTUs, with semi-aquatic frogs having significantly more OTUs when compared to aquatic and arboreal frogs.

Supplementary Figure 6. Results of the Classification and Regression Tree analysis show that ecoregion was the only variable that explained the variance in Faith's Phylogenetic Diversity (PD) among our skin microbiome samples. Samples collected from the Crosstimbers ecoregion were less diverse when compared to the other three ecoregions. Similarly, Arkansas Valley samples had less diversity than those from Central Great Plains and South Central Plains ecoregions.

Supplementary Figure 7. Beta diversity comparisons based on Unweighted-Unifrac distances. **(A)** Principal Coordinates Analysis (PCoA) of all samples with point color indicating the host species: orange = *Acris blanchardi*, green = *Hyla chrysoscelis/versicolor*, red = *Hyla cinerea*, blue = *Rana*

catesbeiana, purple = *Rana sphenocephala*, and shapes representing the different host ecological preferences: triangle = arboreal, circle = aquatic, and square = semi-aquatic. **(B)** PCoAs of each host family separately with Hylidae samples on top and Ranidae samples on the bottom. Color indicates the ecoregion where sampling occurred: dark gray = Arkansas Valley and Central Great Plains, yellow = Crosstimbers and South Central Plains.

Supplementary Tables Legends

Supplementary Table 1. Metadata for 179 skin microbiome samples that were collected and extracted. Rows highlighted in grey represent samples that were removed from downstream analyses due to low sequence counts (i.e. < 1,000 reads per sample).

Supplementary Table 2. Sample sizes for the number of skin microbiome samples before and after rarefaction to exclude samples with low sequence number.
