# Supplementary figures and images for: Host ecology drives frog skin microbiome diversity across ecotone in South-Central North America
Source: Front Microbiomes. 2023 Nov 7;2:1286985. doi: 10.3389/frmbi.2023.1286985 (PMC12993627; doi:10.3389/frmbi.2023.1286985)

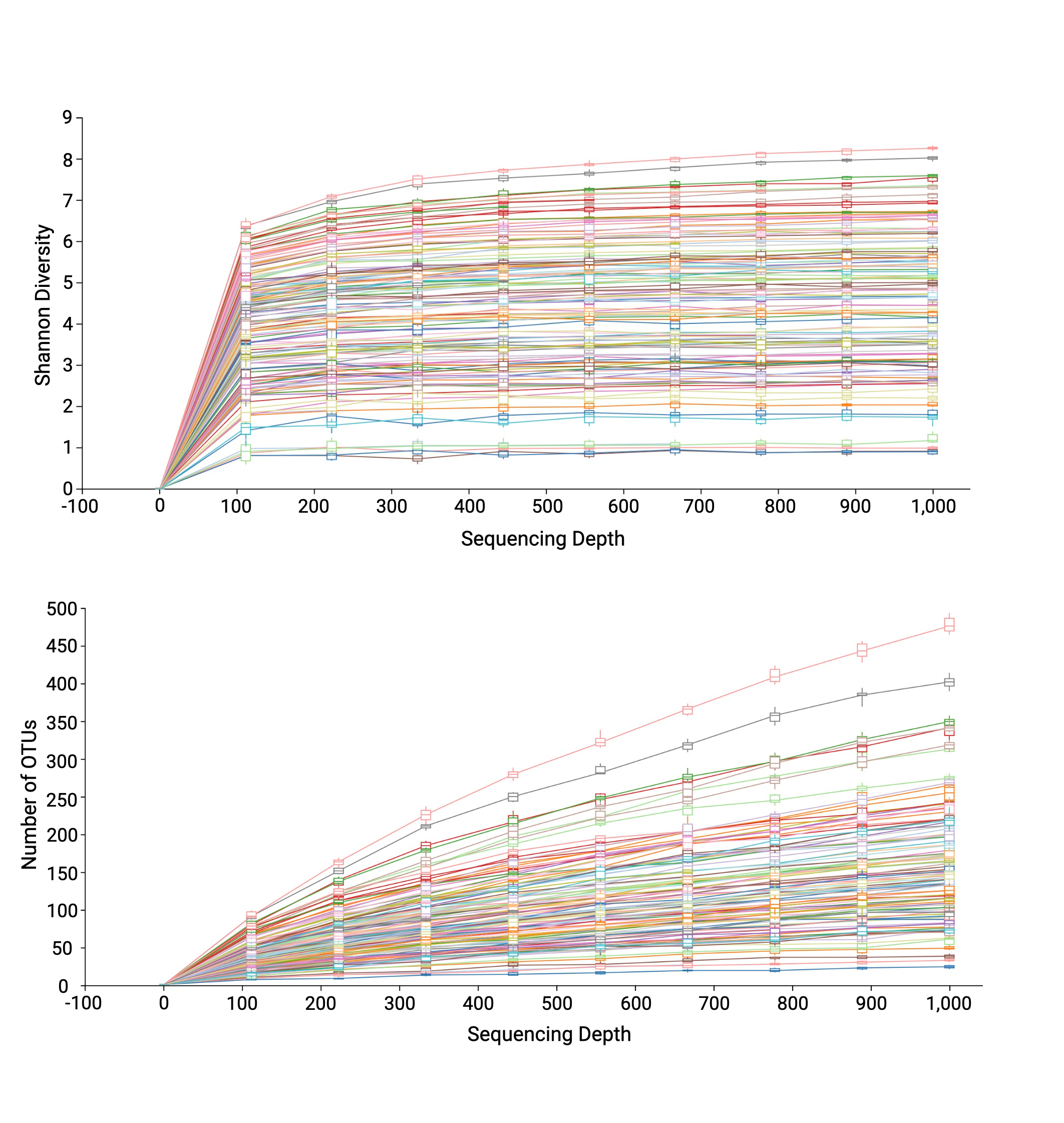

Supplement: Supplementary file 4 [file Image_1.jpeg]

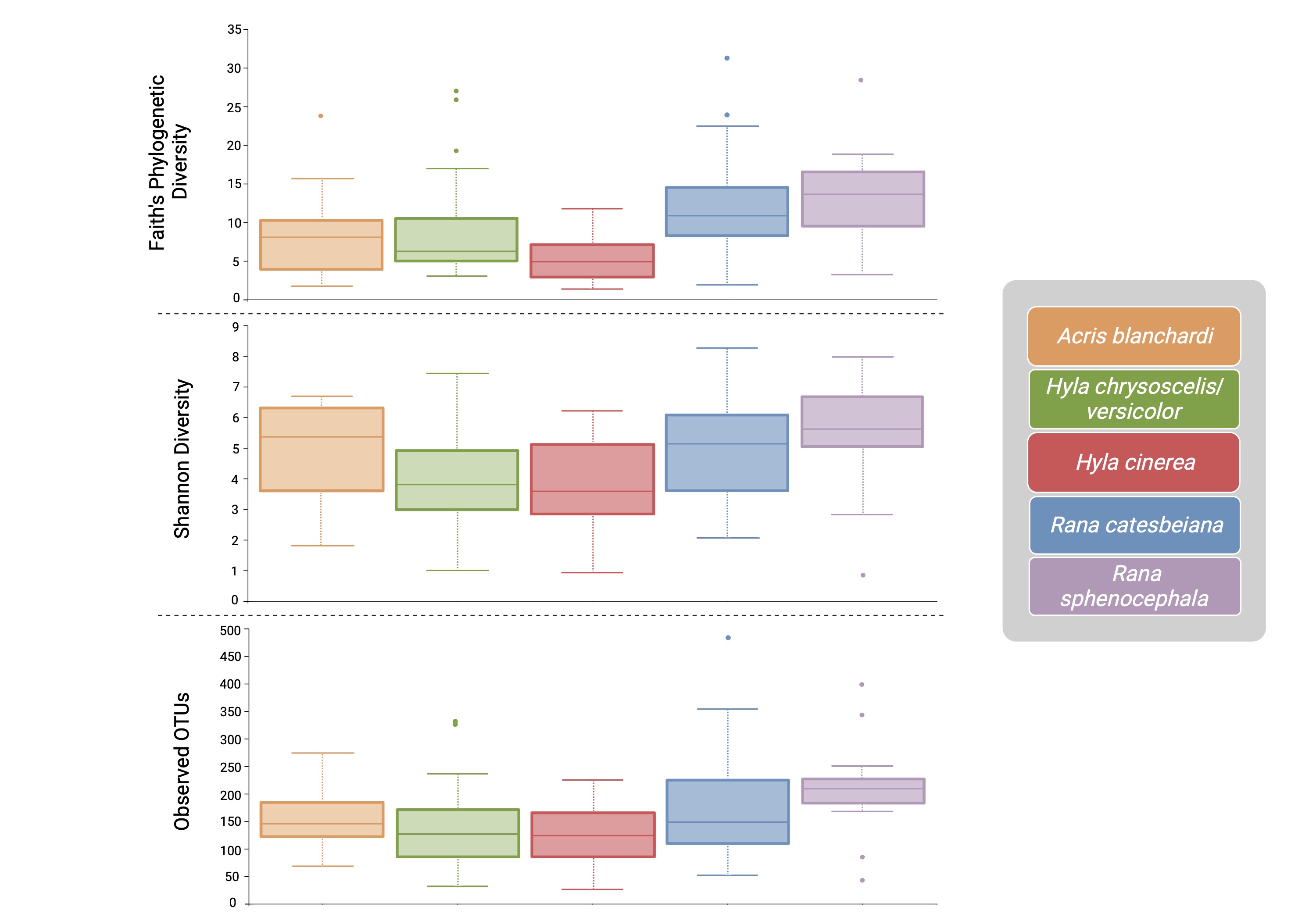

Supplement: Supplementary file 5 [file Image_2.jpeg]

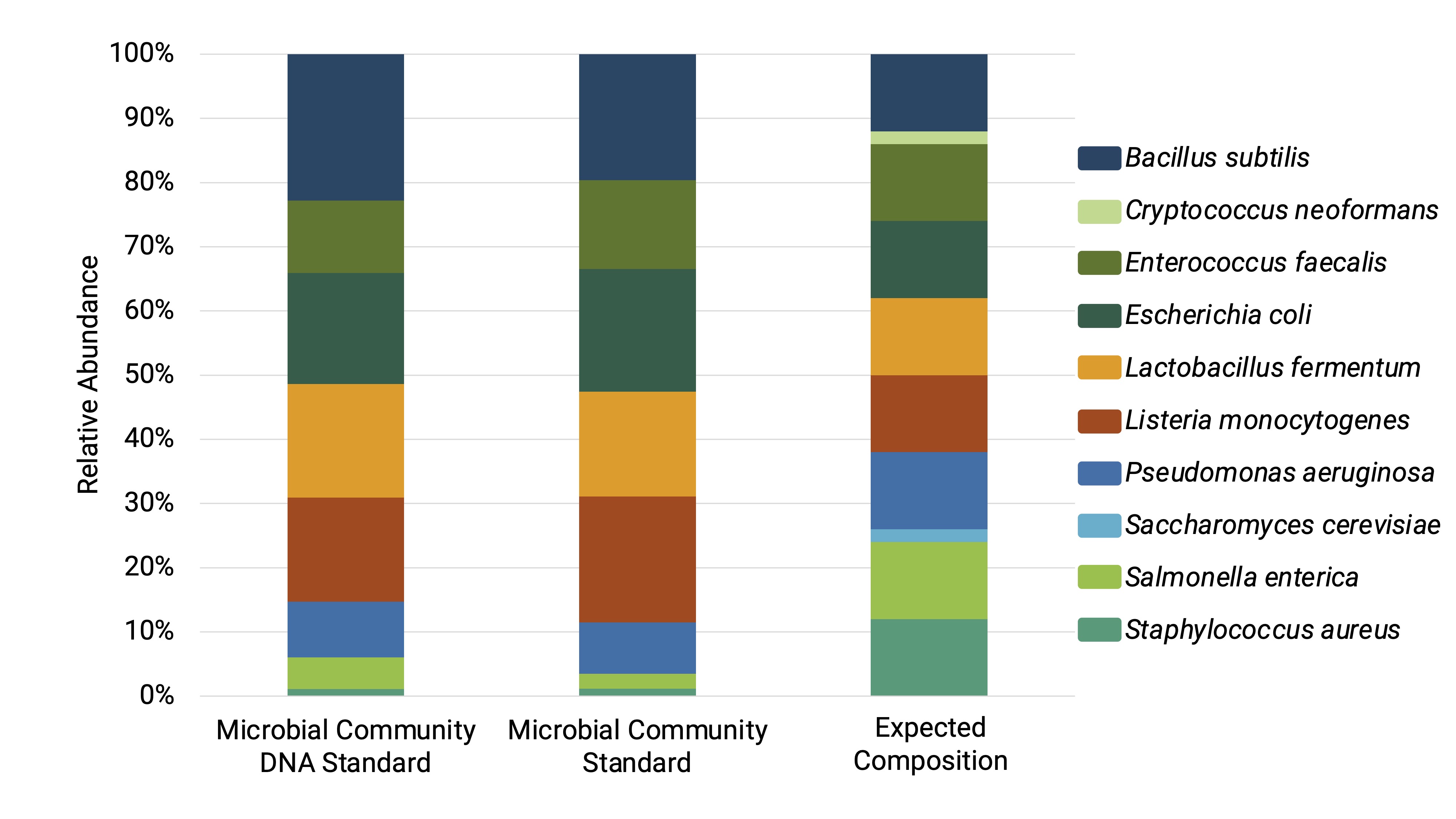

Supplement: Supplementary file 6 [file Image_3.jpeg]

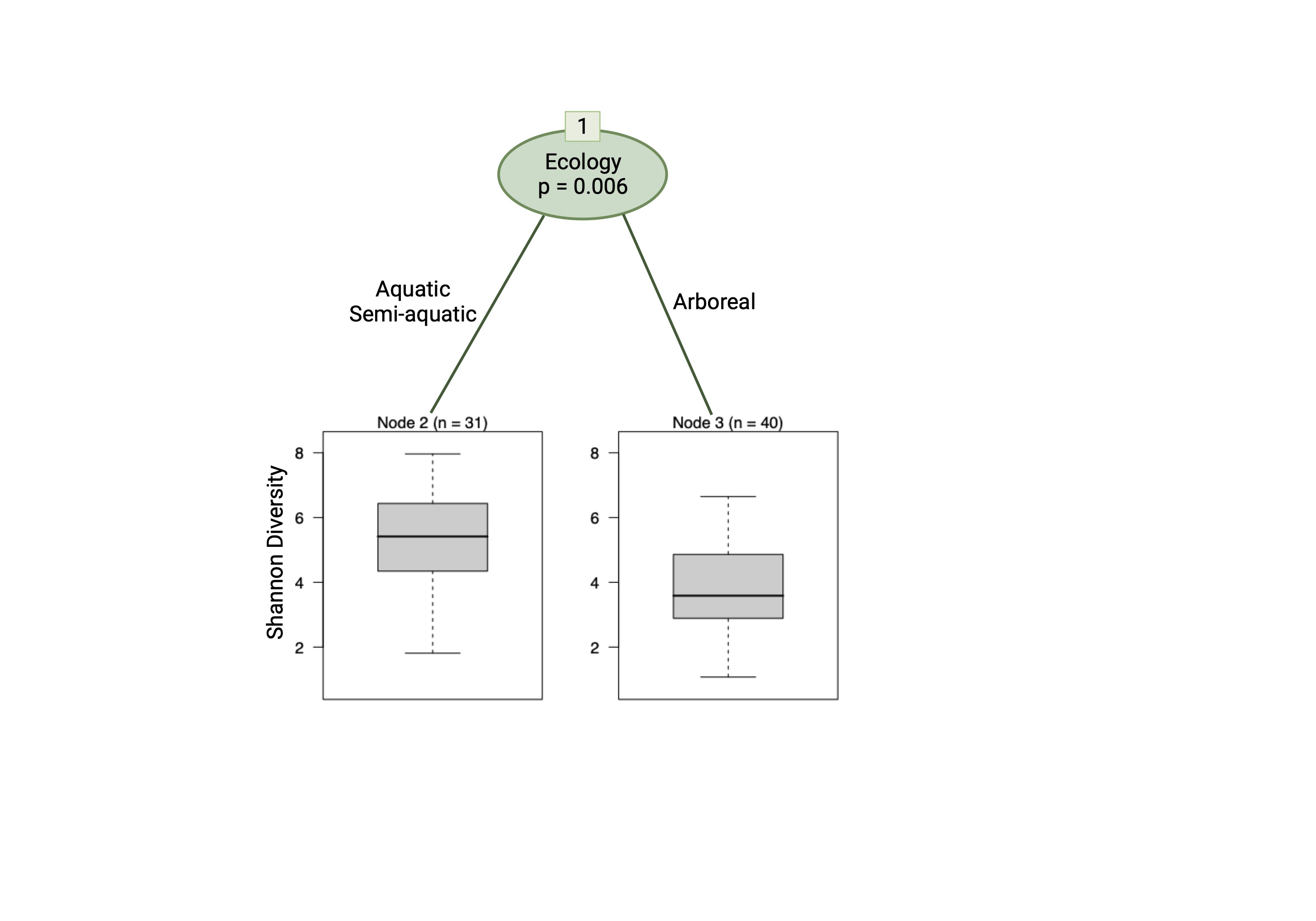

Supplement: Supplementary file 7 [file Image_4.jpeg]

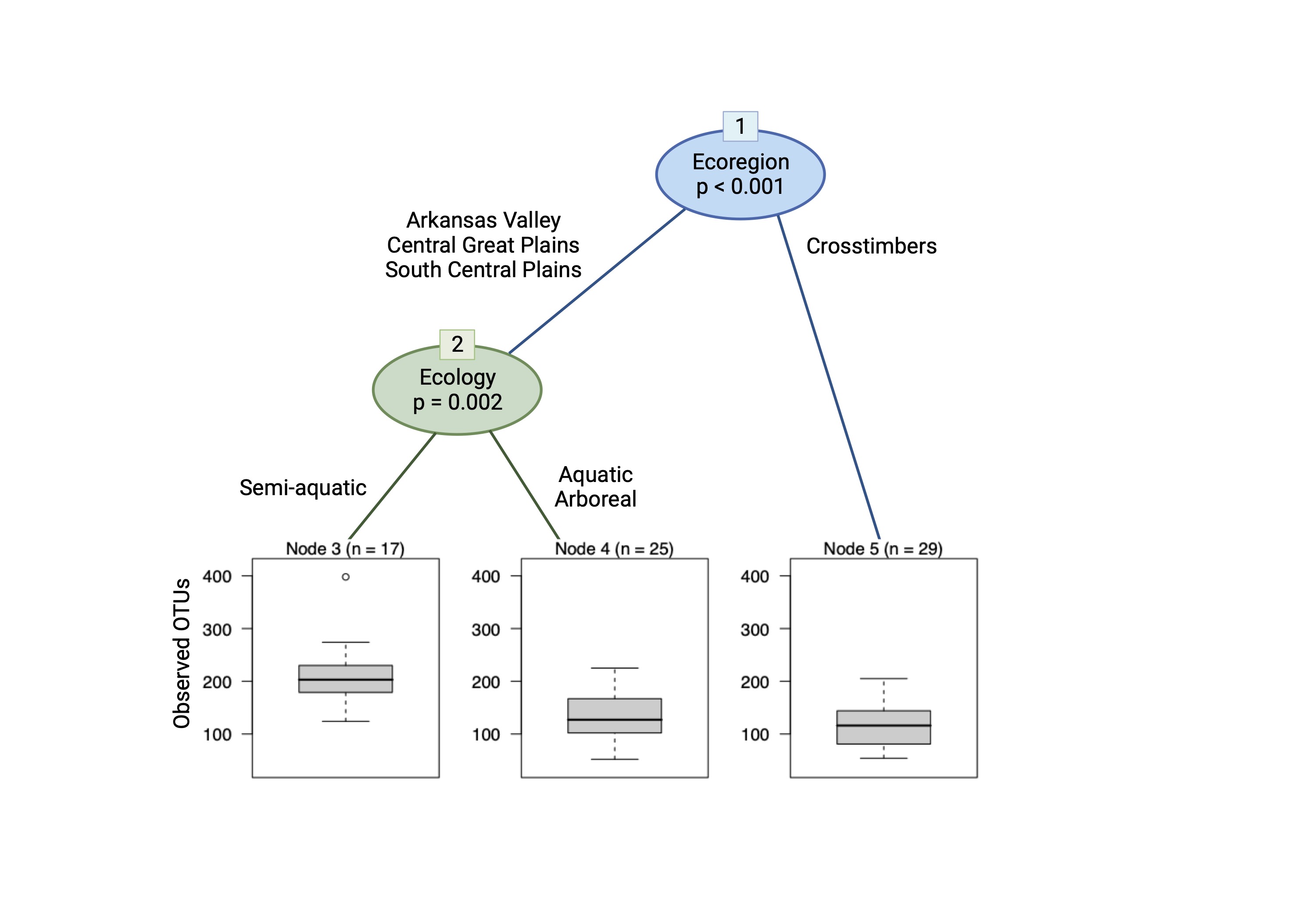

Supplement: Supplementary file 8 [file Image_5.jpeg]

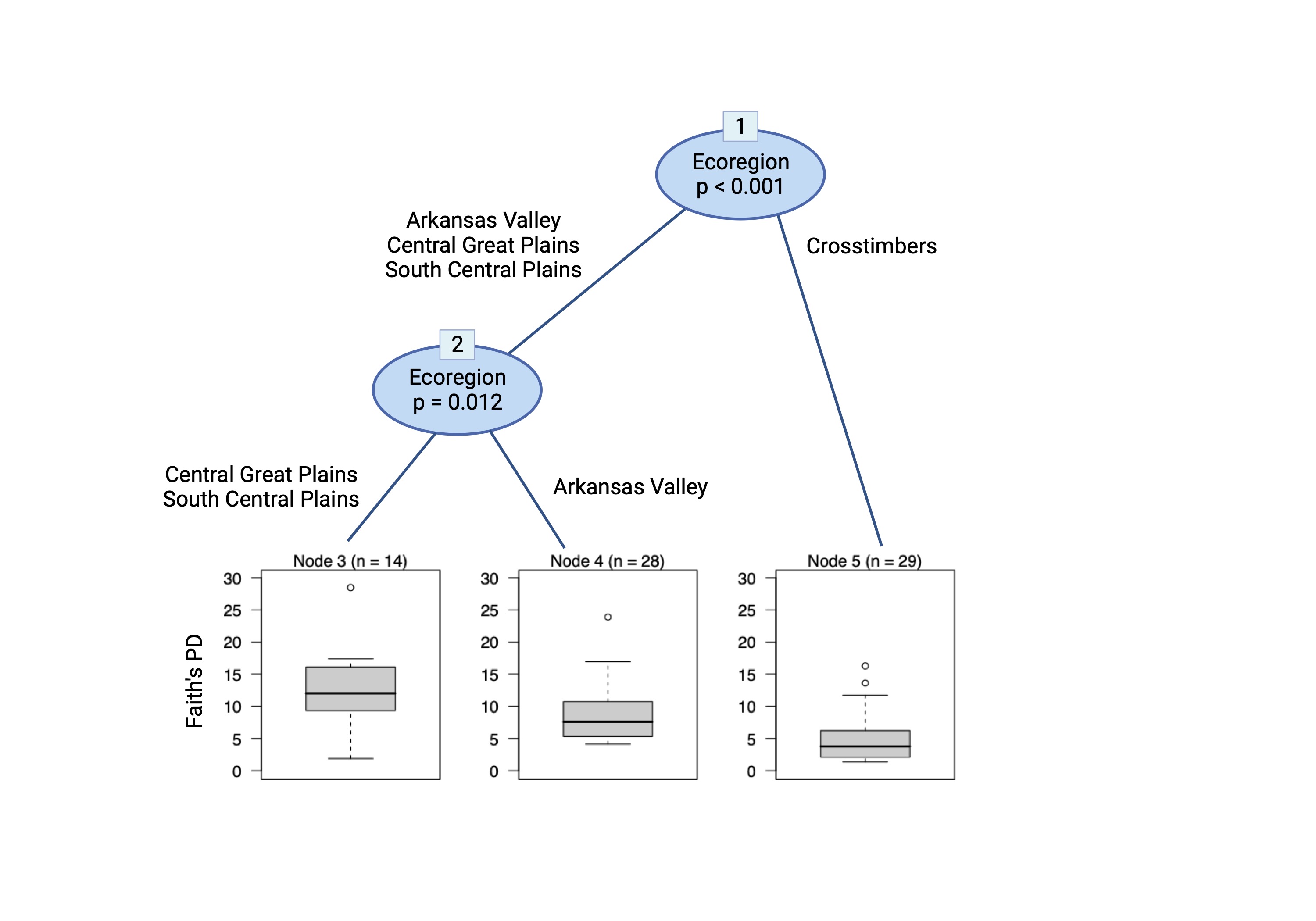

Supplement: Supplementary file 9 [file Image_6.jpeg]

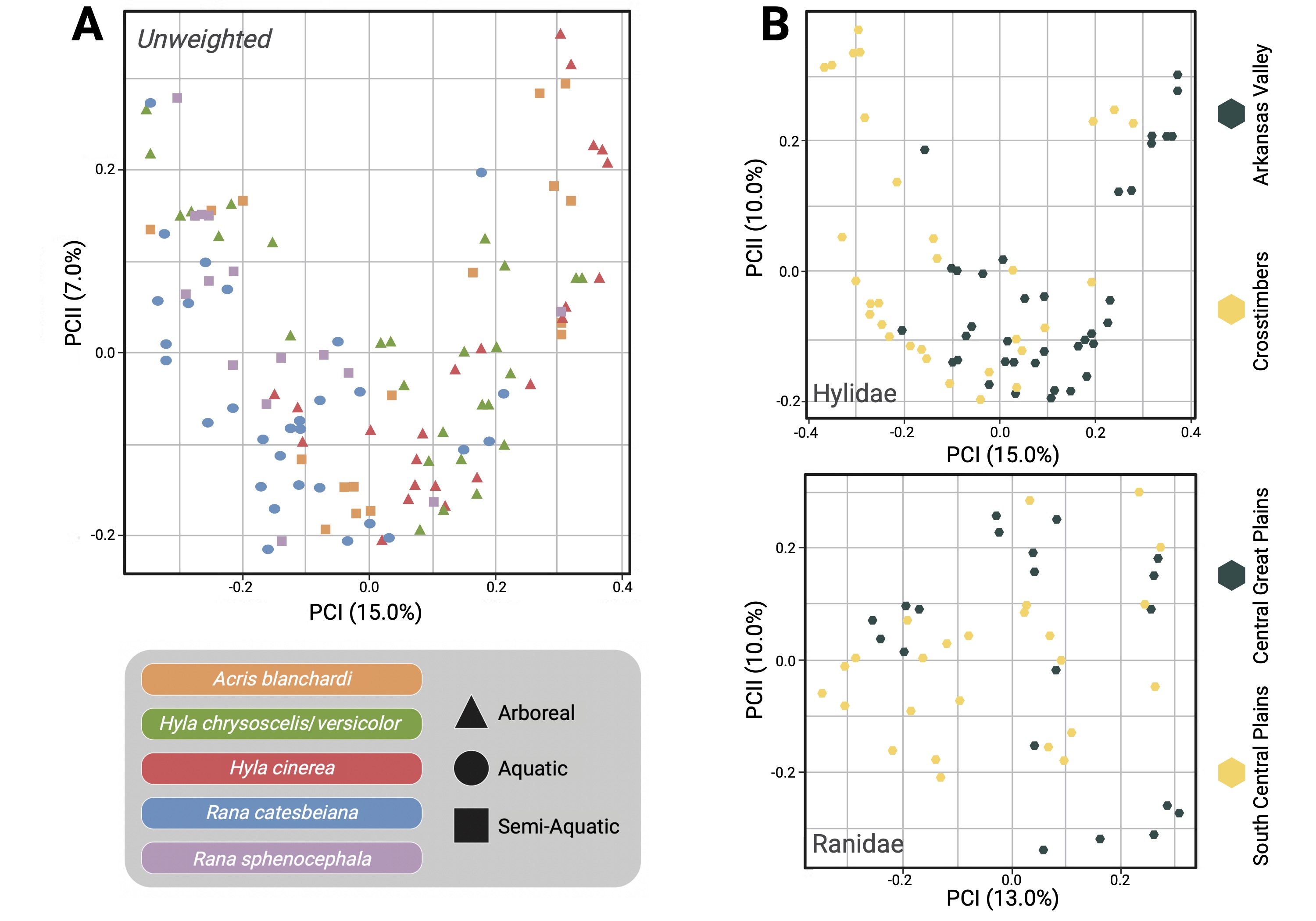

Supplement: Supplementary file 10 [file Image_7.jpeg]
